# Survival Analysis Part I: Basic concepts and first analyses

**DOI:** 10.1038/sj.bjc.6601118

**Published:** 2003-07-15

**Authors:** T G Clark, M J Bradburn, S B Love, D G Altman

**Affiliations:** 1 Cancer Research UK/NHS Centre for Statistics in Medicine, Institute of Health Sciences, University of Oxford, Old Road, Oxford OX3 7LF, UK

**Keywords:** survival analysis, statistical methods, Kaplan-Meier

## Introduction

In many cancer studies, the main outcome under assessment is the time to an event of interest. The generic name for the time is *survival time*, although it may be applied to the time ‘survived’ from complete remission to relapse or progression as equally as to the time from diagnosis to death. If the event occurred in all individuals, many methods of analysis would be applicable. However, it is usual that at the end of follow-up some of the individuals have not had the event of interest, and thus their true time to event is unknown. Further, survival data are rarely Normally distributed, but are skewed and comprise typically of many early events and relatively few late ones. It is these features of the data that make the special methods called *survival analysis* necessary.

This paper is the first of a series of four articles that aim to introduce and explain the basic concepts of survival analysis. Most survival analyses in cancer journals use some or all of Kaplan–Meier (KM) plots, logrank tests, and Cox (proportional hazards) regression. We will discuss the background to, and interpretation of, each of these methods but also other approaches to analysis that deserve to be used more often. In this first article, we will present the basic concepts of survival analysis, including how to produce and interpret survival curves, and how to quantify and test survival differences between two or more groups of patients. Future papers in the series cover multivariate analysis and the last paper introduces some more advanced concepts in a brief question and answer format. More detailed accounts of these methods can be found in books written specifically about survival analysis, for example, Collett (1994), Parmar and Machin (1995) and Kleinbaum (1996). In addition, individual references for the methods are presented throughout the series. Several introductory texts also describe the basis of survival analysis, for example, Altman (2003) and Piantadosi (1997).

## TYPES OF ‘EVENT’ IN CANCER STUDIES

In many medical studies, time to death is the event of interest. However, in cancer, another important measure is the time between response to treatment and recurrence or relapse-free survival time (also called disease-free survival time). It is important to state what the event is and when the period of observation starts and finishes. For example, we may be interested in relapse in the time period between a confirmed response and the first relapse of cancer.

## CENSORING MAKES SURVIVAL ANALYSIS DIFFERENT

The specific difficulties relating to survival analysis arise largely from the fact that only some individuals have experienced the event and, subsequently, survival times will be unknown for a subset of the study group. This phenomenon is called censoring and it may arise in the following ways: (a) a patient has not (yet) experienced the relevant outcome, such as relapse or death, by the time of the close of the study; (b) a patient is lost to follow-up during the study period; (c) a patient experiences a different event that makes further follow-up impossible. Such censored survival times underestimate the true (but unknown) time to event. Visualising the survival process of an individual as a time-line, their event (assuming it were to occur) is beyond the end of the follow-up period. This situation is often called *right censoring*. Censoring can also occur if we observe the presence of a state or condition but do not know where it began. For example, consider a study investigating the time to recurrence of a cancer following surgical removal of the primary tumour. If the patients were examined 3 months after surgery to determine recurrence, then those who had a recurrence would have a survival time that was *left censored* because the actual time of recurrence occurred less than 3 months after surgery. Event time data may also be *interval censored*, meaning that individuals come in and out of observation. If we consider the previous example and patients are also examined at 6 months, then those who are disease free at 3 months and lost to follow-up between 3 and 6 months are considered interval censored. Most survival data include right censored observations, but methods for interval and left censored data are available (Hosmer and Lemeshow, 1999). In the remainder of this paper, we will consider right censored data only.

In general, the feature of censoring means that special methods of analysis are needed, and standard graphical methods of data exploration and presentation, notably scatter diagrams, cannot be used.

## ILLUSTRATIVE STUDIES

### Ovarian cancer data

**Figure 1:**
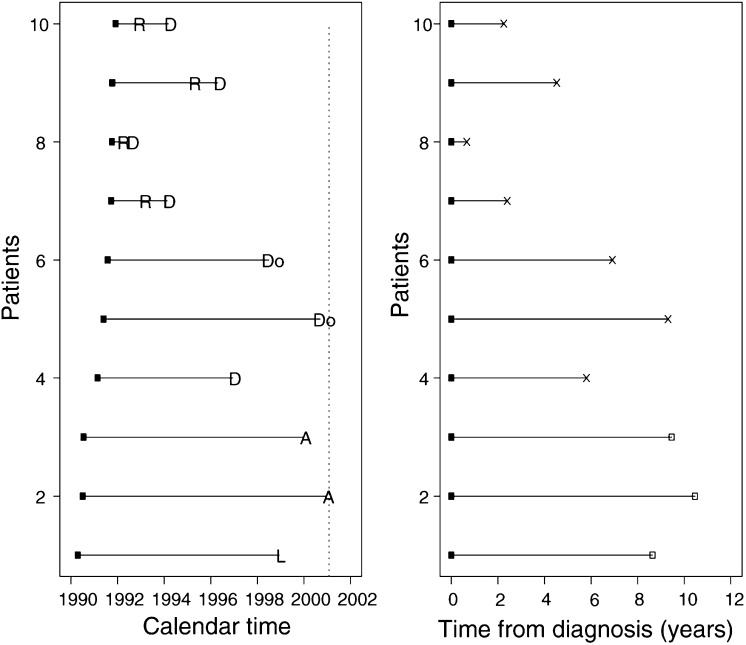
Converting calendar time in the ovarian cancer study to a survival analysis format. Dashed vertical line is the date of the last follow-up, R=relapse, D=death from ovarian cancer, Do=death from other cause, A=attended last clinic visit (alive), L=loss to follow-up, X=death, □=censored.

This data set relates to 825 patients diagnosed with primary epithelial ovarian carcinoma between January 1990 and December 1999 at the Western General Hospital in Edinburgh. Follow-up data were available up until the end of December 2000, by which time 550 (75.9%) had died (Clark *et al*, 2001). Figure 1

shows data from 10 patients diagnosed in the early 1990s and illustrates how patient profiles in calendar time are converted to time to event (death) data. Figure 1 (left) shows that four patients had a nonfatal relapse, one was lost to follow-up, and seven patients died (five from ovarian cancer). In the other plot, the data are presented in the format for a survival analysis where all-cause mortality is the event of interest. Each patient's ‘survival’ time has been plotted as the time from diagnosis. It is important to note that because overall mortality is the event of interest, nonfatal relapses are ignored, and those who have not died are considered (right) censored. Figure 1 (right) is specific to the outcome or event of interest. Here, death from any cause, often called overall survival, was the outcome of interest. If we were interested solely in ovarian cancer deaths, then patients 5 and 6 – those who died from nonovarian causes – would be censored. In general, it is good practice to choose an end-point that cannot be misclassified. All-cause mortality is a more robust end-point than a specific cause of death. If we were interested in time to relapse, those who did not have a relapse (fatal or nonfatal) would be censored at either the date of death or the date of last follow-up.

### Lung cancer clinical trial data

These data originate from a phase III clinical trial of 164 patients with surgically resected (non-small cell) lung cancer, randomised between 1979 and 1985 to receive radiotherapy either with or without adjuvant combination platinum-based chemotherapy (Lung Cancer Study Group, 1988; Piantadosi, 1997). For the purposes of this series, we will focus on the time to first relapse (including death from lung cancer). Table 1

**Table 1:** A sample of times (days) to relapse among patients randomised to receive radiotherapy with or without adjuvant chemotherapy

Radiotherapy (*n*=86)	18, 23^a^, 25, 27, 28, 30, 36, 45, 55, 56, 57, 57, 57, 59, 62, …,
	2252^a^, 2286^a^, 2305^a^, 2318^a^, 2940^a^
Radiotherapy+CAP (*n*=78)	9, 22, 35, 53, 76, 81, 94, 97, 103, 114, 115, 126, 147, 154, …,
	2220^a^, 2375, 2566, 2875^b^, 3067^b^

CAP=cytoxan, doxorubicin and platinum-based chemotherapy.

a Lost to follow-up and considered censored.

b Relapse-free at time of analysis and considered censored.

gives the time of the earliest 15 and latest five relapses for each treatment group, where it can be seen that some patients were alive and relapse-free at the end of the study. The relapse proportions in the radiotherapy and combination arms were 81.4% (70 out of 86) and 69.2% (54 out of 78), respectively. However, these figures are potentially misleading as they ignore the duration spent in remission before these events occurred.

## SURVIVAL AND HAZARD

Survival data are generally described and modelled in terms of two related probabilities, namely *survival* and *hazard*. The survival probability (which is also called the survivor function) *S*(*t*) is the probability that an individual survives from the time origin (e.g. diagnosis of cancer) to a specified future time *t*. It is fundamental to a survival analysis because survival probabilities for different values of *t* provide crucial summary information from time to event data. These values describe directly the survival experience of a study cohort.

The hazard is usually denoted by *h*(*t*) or *λ*(*t*) and is the probability that an individual who is under observation at a time *t* has an event at that time. Put another way, it represents the instantaneous event rate for an individual who has already survived to time *t*. Note that, in contrast to the survivor function, which focuses on not having an event, the hazard function focuses on the event occurring. It is of interest because it provides insight into the conditional failure rates and provides a vehicle for specifying a survival model. In summary, the hazard relates to the incident (current) event rate, while survival reflects the cumulative non-occurrence.

## KAPLAN–MEIER SURVIVAL ESTIMATE

The survival probability can be estimated nonparametrically from observed survival times, both censored and uncensored, using the KM (or product-limit) method (Kaplan and Meier, 1958). Suppose that *k* patients have events in the period of follow-up at distinct times *t*_1_<*t*_2_<*t*_3_<*t*_4_<*t*_5_<⋯<*t_k_*. As events are assumed to occur independently of one another, the probabilities of surviving from one interval to the next may be multiplied together to give the cumulative survival probability. More formally, the probability of being alive at time *t_j_*, *S*(*t_j_*), is calculated from *S*(*t_j__−1_*) the probability of being alive at *t_j__−1_*, *n_j_* the number of patients alive just before *t_j_*, and *d_j_* the number of events at *t_j_*, by


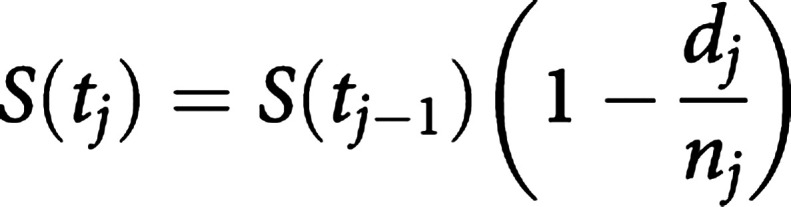


where *t*_0_=0 and *S*(0)=1. The value of *S*(*t*) is constant between times of events, and therefore the estimated probability is a step function that changes value only at the time of each event. This estimator allows each patient to contribute information to the calculations for as long as they are known to be event-free. Were every individual to experience the event (i.e. no censoring), this estimator would simply reduce to the ratio of the number of individuals events free at time *t* divided by the number of people who entered the study.

Confidence intervals for the survival probability can also be calculated. The KM *survival curve*, a plot of the KM survival probability against time, provides a useful summary of the data that can be used to estimate measures such as median survival time. The large skew encountered in the distribution of most survival data is the reason that the mean is not often used.

### Survival analysis of the lung cancer trial

Table 2

**Table 2:** Calculation of the relapse-free survival probability for patients in the lung cancer trial

**Radiotherapy (*n*=86)**		**Radiotherapy+CAP (*n*=78)**	
**Survival times (days)**	**Kaplan–Meier survivor function *S*(*t*)**	**Survival times (days)**	**Kaplan–Meier survivor function *S*(*t*)**
18	1 × (1-1/86)=0.988	9	1 × (1-1/78)=0.987
23^a^	S(18) × (1-0/85)=0.988	22	S(18) × (1-1/77)=0.974
25	S(23) × (1-1/84)=0.977	35	S(22) × (1-1/76)=0.962
27	S(25) × (1-1/83)=0.965	53	S(35) × (1-1/75)=0.949
28	S(27) × (1-1/82)=0.953	76	S(53) × (1-1/74)=0.936
30	S(28) × (1-1/81)=0.941	81	S(76) × (1-1/73)=0.923
36	S(30) × (1-1/80)=0.930	94	S(81) × (1-1/72)=0.910
45	S(36) × (1-1/79)=0.918	97	S(94) × (1-1/71)=0.897
55	S(45) × (1-1/78)=0.906	103	S(97) × (1-1/70)=0.885
56	S(55) × (1-1/77)=0.894	114	S(103) × (1-1/69)=0.872
57	S(56) × (1-3/76)=0.859	115	S(114) × (1-1/68)=0.859
57	S(56) × (1-3/76)=0.859	121^a^	S(115) × (1-0/67)=0.859
57	S(56) × (1-3/76)=0.859	126	S(121) × (1-1/66)=0.846
59	S(57) × (1-1/73)=0.847	147	S(126) × (1-1/65)=0.833
62	S(59) × (1-1/72)=0.835	154	S(147) × (1-1/64)=0.820
	⋮		⋮
2252^a^	S(2209) × (1-0/5)=0.115	2220^a^	S(2218) × (1-0/5)=0.273
2286^a^	S(2286) × (1-0/4)=0.115	2375	S(2220) × (1-0/4)=0.205
2305^a^	S(2305) × (1-0/3)=0.115	2566	S(2375) × (1-0/3)=0.137
2318^a^	S(2318) × (1-0/2)=0.115	2875^b^	S(2566) × (1-0/2)=0.137
2940^a^	S(2940) × (1-0/1)=0.115	3067^b^	S(2875) × (1-0/1)=0.137

*S*(0)=1, (CAP=cytoxan, doxorubicin and platinum-based chemotherapy.)

a Lost to follow-up and considered censored.

b Relapse-free at time of analysis and considered censored.

shows the essential features of the KM survival probability. The estimator at any point in time is obtained by multiplying a sequence of conditional survival probabilities, with the estimate being unchanged between subsequent event times. For example, the probability of a member of the radiotherapy alone treatment group surviving (relapse-free) 45 days is the probability of surviving the first 36 days multiplied by the probability of then surviving the interval between 36 and 45 days. The latter is a *conditional* probability as the patient needs to have survived the first period of time in order to remain in the study for the second. The KM estimator utilises this fact by dividing the time axis up according to event times and estimating the event probability in each division, from which the overall estimate of the survivorship is drawn.

Figure 2

**Figure 2:**
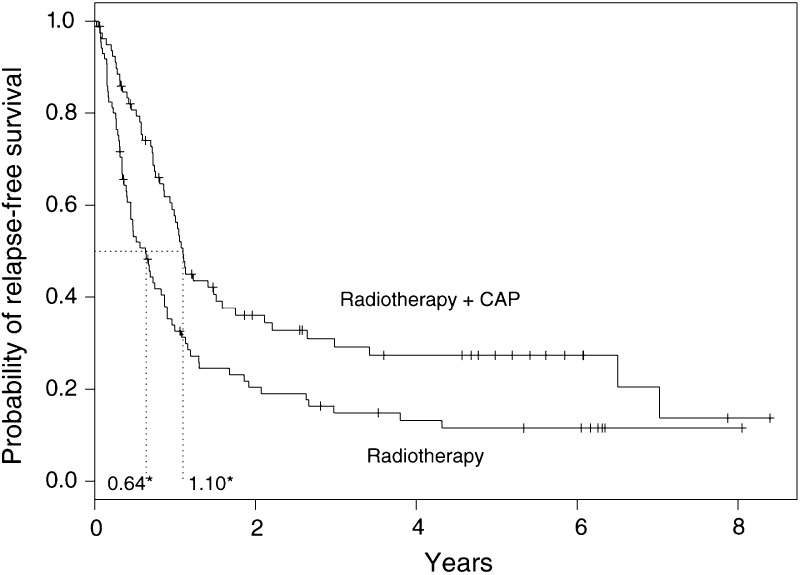
Relapse-free survival curves for the lung cancer trial. ^*^ Median relapse-free survival time for each arm, + censoring times, CAP=cytoxan, doxorubicin and platinum-based chemotherapy.

shows the survival probabilities for the two treatment groups in the conventional KM graphical display. The median survival times for each group are shown and represent the time at which *S*(*t*) is 0.5. The combination group has a median survival time of 402 days (1.10 years), as opposed to 232 days (0.64 years) in the radiotherapy alone arm, providing some evidence of a chemotherapy treatment benefit. Other survival time percentiles may be read directly from the plot or (more accurately) from a full version of Table 2. There appears to be a survival advantage in the combination therapy group, but whether this difference is statistically significant requires a formal statistical test, a subject that is discussed later.

### Survival function of the ovarian data

**Figure 3:**
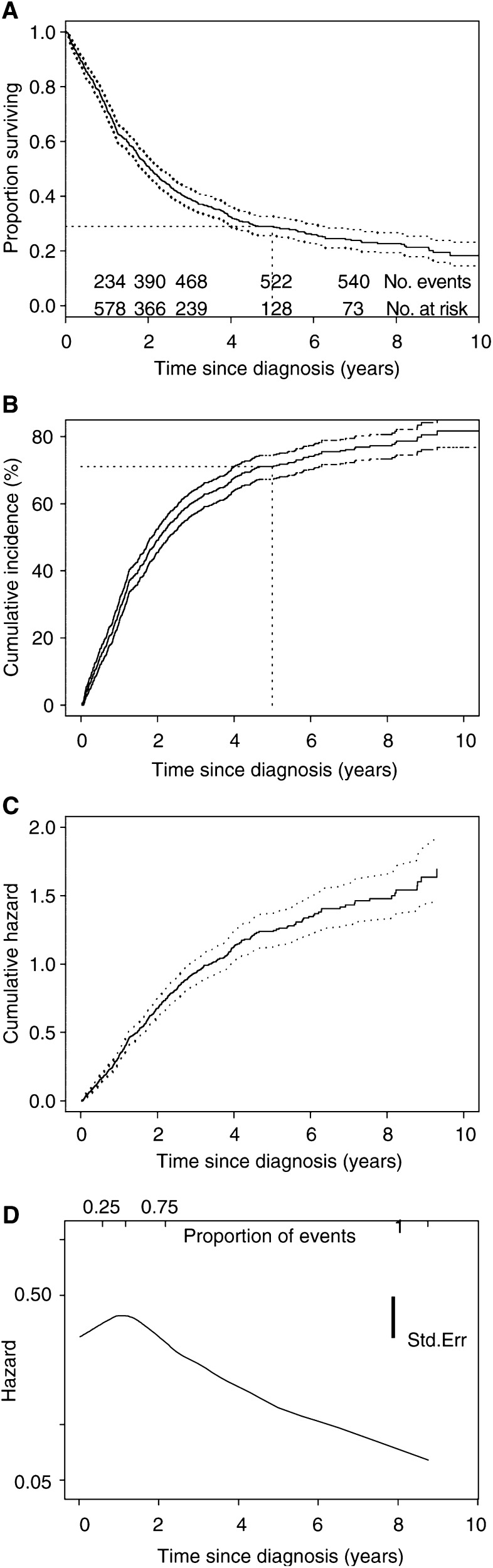
Survival and cumulative hazard curves with 95% CIs for the ovarian cancer study. Std.Err=standard error. (**A**) Kaplan–Meier survivor function, (**B**) cumulative incidence curve, (**C**) cumulative hazard function, (**D**) hazard function (smoothed).

The KM survival curve of the ovarian cancer data is shown in Figure 3A

. The steep decline in the early years indicates poor prognosis from the disease. This is also indicated by changes in the cumulative number of events and number at risk. Specifically, of the 825 women diagnosed with ovarian cancer, about a third had died within the first year, accounting for 43% of the total deaths as recorded by the last date of follow-up. The number lost to follow-up can be deduced from the total number in the cohort and the cumulative number of events and number at risk.

The 95% confidence limits of the survivor function are shown. In practice, there are usually patients who are lost to follow-up or alive at the end of follow-up, and confidence limits are often wide at the tail of the curve, making meaningful interpretations difficult. Thus, it may be sensible to curtail plots before the end of follow-up on the *x*-axis (Pocock *et al*, 2002). Curtailing of the *y*-axis, a common practice for diseases or events of low incidence, should not be performed. Instead, the incidence of death curve, or 1−*S*(*t*), (Figure 3B) may be presented (Pocock *et al*, 2002). The cumulative incidence at a time point is simply one minus the survival probability. For example, Figure 3A shows how the 5-year survival of 0.29 (29%) is calculated, and could also be read from Figure 3B as a cumulative incidence of 71% for the first 5 years.

## HAZARD AND CUMULATIVE HAZARD

There is a clearly defined relationship between *S*(*t*) and *h*(*t*), which is given by the calculus formula:


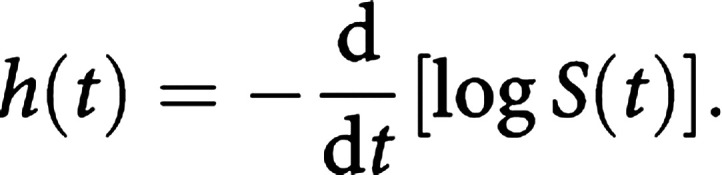


The formula is unimportant for routine survival analyses as it is incorporated into most statistical computer packages. The point here is simply that if either *S*(*t*) or *h*(*t*) is known, the other is automatically determined. Consequently, either can be the basis of statistical analysis.

Unfortunately, unlike *S*(*t*) there is no simple way to estimate *h*(*t*). Instead, a quantity called the *cumulative hazard H*(*t*) is commonly used. This is defined as the integral of the hazard, or the area under the hazard function between times 0 and *t*, and differs from the log-survivor curve only by sign, that is *H*(*t*)=−log[*S*(*t*)]. The interpretation of *H*(*t*) is difficult, but perhaps the easiest way to think of *H*(*t*) is as the cumulative force of mortality, or the number of events that would be expected for each individual by time *t* if the event were a repeatable process. *H*(*t*) is used an intermediary measure for estimating *h*(*t*) and as a diagnostic tool in assessing model validity. A simple nonparametric method for estimating *H*(*t*) is the Nelson-Aalen estimator (Hosmer, 1999), from which it is possible to derive an estimate of *h*(*t*) by applying a kernel smoother to the increments (Ramlau-Hansen, 1983). Cox (1979) suggests another method to estimate the hazard based on order statistics but similar in spirit to the previous method.

**Figure 4:**
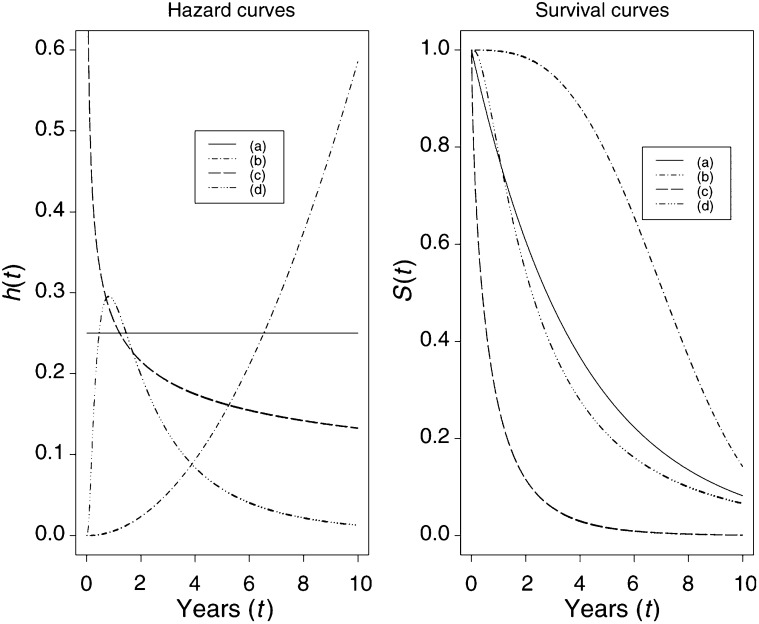
Relationships between (parametric) hazard and survival curves: (a) constant hazard (e.g. healthy persons), (b) increasing Weibull (e.g. leukaemia patients), (c) decreasing Weibull (e.g. patients recovering from surgery), (d) increasing and then decreasing log-normal (e.g. tuberculosis patients).

Another approach to estimating the hazard is to assume that the survival times follow a specific mathematical distribution. Figure 4

shows the relationship between four parametrically specified hazards and the corresponding survival probabilities. It illustrates a constant hazard rate over time (which is analogous to an exponential distribution of survival times), strictly increasing/decreasing hazard rates based on a Weibull model, and a combination of decreasing and increasing hazard rates using a log-Normal model. These curves are illustrative examples and other shapes are possible. The specification of hazards using fully parametric distributions is an important and under-utilised modelling technique that will be discussed in subsequent papers.

### Hazard function in the ovarian data

Figure 3C shows the cumulative hazard for the ovarian cancer data. The hazard is shown in Figure 3D. As the hazard function is generally very erratic, it is customary to fit a smooth curve to enable the underlying shape to be seen. Figure 3D shows that the (instantaneous) risk of death appears to be high in the first year after diagnosis and decreases afterwards. This observation corresponds to the steeply descending survival probability (Figure 3A) and marked increase in cumulative incidence (Figure 3B) in the first year. The *y*-axis is difficult to interpret for the hazard and the cumulative hazard, but the decreasing shape of the hazard may be consistent with a decreasing Weibull's model (see Figure 4).

## NONPARAMETRIC TESTS COMPARING SURVIVAL

Survival in two or more groups of patients can be compared using a nonparametric test. The logrank test (Peto *et al*, 1977) is the most widely used method of comparing two or more survival curves. The groups may be treatment arms or prognostic groups (e.g. FIGO stage). The method calculates at each event time, for each group, the number of events one would expect since the previous event if there were no difference between the groups. These values are then summed over all event times to give the total expected number of events in each group, say *E_i_* for group *i*. The logrank test compares observed number of events, say *O_i_* for treatment group *i*, to the expected number by calculating the test statistic


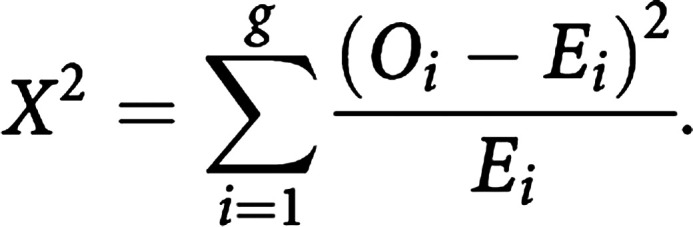


This value is compared to a *χ*^2^ distribution with (*g*−1) degrees of freedom, where *g* is the number of groups. In this manner, a *P*-value may be computed to calculate the statistical significance of the differences between the complete survival curves.

If the groups are naturally ordered, a more appropriate test is to consider the possibility that there is a trend in survival across them, for example, age groups or stages of cancer. Calculating *O_i_* and *E_i_* for each group on the basis that survival may increase or decrease across the groups results in a more powerful test. For the new *O_i_* and *E_i_*, the test statistic for trend is compared with the *χ*^2^ distribution with one degree of freedom (Collett, 1994).

When only two groups are compared, the logrank test is testing the null hypothesis that the ratio of the hazard rates in the two groups is equal to 1. The hazard ratio (HR) is a measure of the relative survival experience in the two groups and may be estimated by


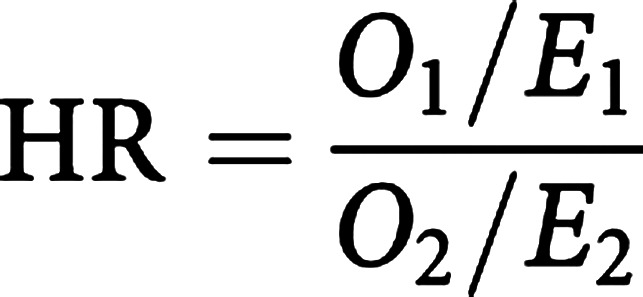


where *O_i_/E_i_* is the estimated relative (excess) hazard in group *i*. A confidence interval (CI) for the HR can be calculated (Collett, 1994). The HR has a similar interpretation of the strength of effect as a risk ratio. An HR of 1 indicates no difference in survival. In practice, it is better to estimate HRs using a regression modelling technique, such as Cox regression, as described in the next article.

Other nonparametric tests may be used to compare groups in terms of survival (Collett, 1994). The logrank test is so widely used that the reason for any other method should be stated in the protocol of the study. Alternatives include methods to compare median survival times, but comparing confidence intervals for each group is not recommended (Altman and Bland, 2003). The logrank method is considered more robust (Hosmer and Lemeshow, 1999), but the lack of an accompanying effect size to compliment the *P*-value it provides is a limitation.

### Survival differences in the lung cancer trial

We have already seen that median survival is greater in the combination treatment arm. Table 3

**Table 3:** Differences in (relapse-free) survival in the lung cancer trial

	**Radiotherapy (*n*=86)**	**Radiotherapy+CAP (*n*=78)**
Number of relapses (*O*_*i*_)	70	54
Median survival time(years) (95% CI)	0.64 (0.45–0.87)	1.10 (0.96–1.59)
Expected number of relapses (*E*_*i*_)	53.4	70.6
Hazard ratio (95% CI)	0.58 (0.41–0.83)	
Logrank test	*χ*^2^=9.1, 1 df, *P*<0.002	

df=degree of freedom: CAP=cytoxan, doxorubicin and platinum-based chemotherapy.

provides information about (relapse-free) survival differences between the trial arms. A test of differences between median survival times in the groups is indicative of a difference in survival (*P*<0.01). The number of relapses observed among patients treated with radiotherapy+CAP (cytoxan, doxorubin and platinum-based chemotherapy) and radiotherapy alone were 54 and 70, respectively. Using the logrank method, the expected number of relapses for each group were 70.6 and 53.4, respectively. Thus, the logrank test yields a *χ*^2^ value of 9.1 on 1 degree of freedom (*P*<0.002). The HR of 0.58 indicates that there is 42% less risk of relapse at any point in time among patients surviving in the combination treatment group compared with those treated with radiotherapy alone. Overall, there is an indication that the combination treatment is more efficacious than radiotherapy treatment, and may be preventing or delaying relapse.

### Survival differences in the ovarian study

**Figure 5:**
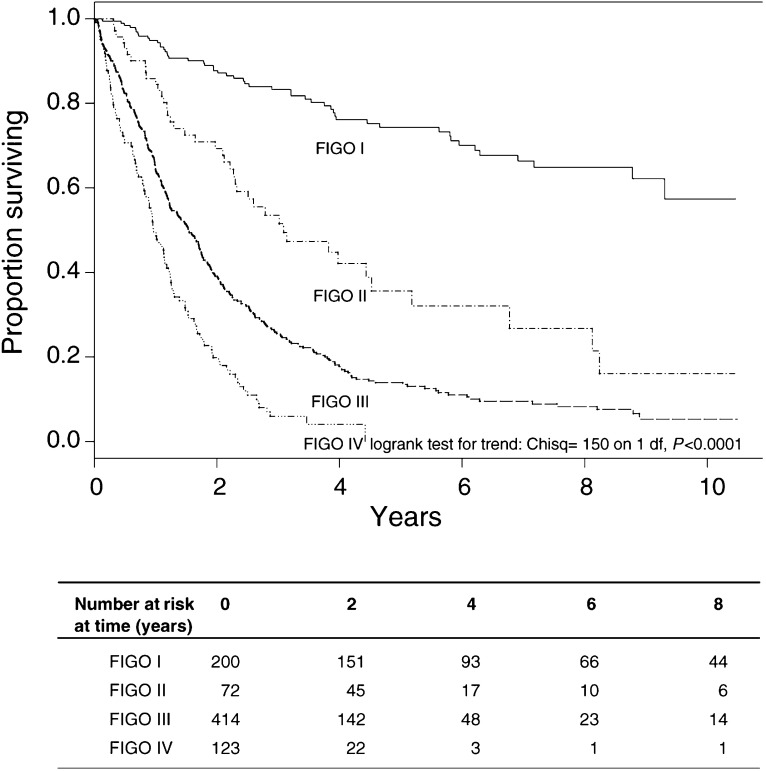
FIGO stage and prognosis in the ovarian study. Chisq=*χ*^2^.

In the ovarian study, we wished to compare the survival between patients with different FIGO stages–an ordinal variable. Figure 5

shows overall survival by FIGO stage. A logrank test of trend is statistically significant (*P*<0.0001), and reinforces the visual impression of prognostic separation and a trend towards better survival when the disease is less advanced.

## SOME KEY REQUIREMENTS FOR THE ANALYSIS OF SURVIVAL DATA

### Uninformative censoring

Standard methods used to analyse survival data with censored observations are valid only if the censoring is ‘noninformative’. In practical terms, this means that censoring carries no prognostic information about subsequent survival experience; in other words, those who are censored because of loss to follow-up at a given point in time should be as likely to have a subsequent event as those individuals who remain in the study. Informative censoring may occur when patients withdraw from a clinical trial because of drug toxicity or worsening clinical condition. Standard methods for survival analysis are not valid when there is informative censoring. However, when the number of patients lost to follow-up is small, very little bias is likely to result from applying methods based on noninformative censoring.

### Length of follow-up

In general, the design of a study will influence how it is analysed. Time to event studies must have sufficient follow-up to capture enough events and thereby ensure there is sufficient power to perform appropriate statistical tests. The proposed length of follow-up for a prospective study will be based primarily on the severity of the disease or prognosis of the participants. For example, for a lung cancer trial a 5-year follow-up would be more than adequate, but this follow-up duration will only give a short- to-medium-term indication of survivorship among breast cancer patients.

An indicator of length of follow-up is the median follow-up time. While this could in theory be given as the median follow-up time of all patients, it is better calculated from follow-up among the individuals with censored data. However, both these methods tend to underestimate follow-up, and a more robust measure is based on the reverse KM estimator (Schemper and Smith, 1996), that is the KM method with the event indicator reversed so that the outcome of interest becomes being censored. In the ovarian cohort example, the median follow-up time of all the patients is 1.7 years, although is influenced by the survival times which were early deaths. The median survival of the censored patients was 3.2 years, but the reverse KM estimate of the median follow-up is 5.3 years (95% CI: 4.7–6.0 years).

### Completeness of follow-up

Each patient who does not have an event can be included in a survival analysis for the period up to the time at which they are censored, but completeness of follow-up is still important. Unequal follow-up between different groups, such as treatment arms, may bias the analysis. A simple count of participants lost to follow-up is one indicator of data incompleteness, but it does not inform us about time lost and another measure has been proposed (Clark *et al*, 2002). In general, disparities in follow-up caused by differential drop-out between arms of a trial or different subgroups in a cohort study need to investigated.

### Cohort effect on survival

In survival analysis, there is an assumption of homogeneity of treatment and other factors during the follow-up period. However, in a long-term observational study of patients of cancer, the case mix may change over the period of recruitment, or there may be an innovation in ancillary treatment. The KM method assumes that the survival probabilities are the same for subjects recruited early and late in the study. On average, subjects with longer survival times would have been diagnosed before those with shorter times, and changes in treatments, earlier diagnosis or some other change over time may lead to spurious results. The assumption may be tested, provided we have enough data to estimate survival probabilities in different subsets of the data and, if necessary, adjusted for by further analyses (see next section).

### Between-centre differences

In a multicentre study, it is important that there is a consistency between the study methods in each centre. For example, diagnostic instruments, such as staging classification, and treatments should be identical. Heterogeneity in case mix among centres can be adjusted for in an analysis (see next section).

## NEED FOR SURVIVAL ANALYSIS ADJUSTING FOR COVARIATES

When comparing treatments in terms of survival, it is often sensible to adjust for patient-related factors, known as covariates or confounders, which could potentially affect the survival time of a patient. For example, suppose that despite the treatment being randomised in the lung cancer trial, older patients were assigned more often to the radiotherapy alone group. This group would have a worse baseline prognosis and so the simple analysis may have underestimated its efficacy compared to the combination treatment, referred to as confounding between treatment and age. Also, we sometimes want to determine the prognostic ability of various factors on overall survival, as in the ovarian study. Figure 5 shows overall survival by FIGO stage, and there is a significant decrease in overall survival with more advanced disease.

Multiple prognostic factors can be adjusted for using multivariate modelling. For example, if those women with early stage disease were younger than those with advanced disease, then the FIGO I and II groups might be surviving longer because of lower age and not because of the effect of FIGO stage. In this case, the FIGO effect is confounded by the effect of age, and a multivariate analysis is required to adjust for the differences in the age distribution. The appropriate analysis is a form of multiple regression, and is the subject of the next paper in this series.

## SUMMARY

Survival analysis is a collection of statistical procedures for data analysis where the outcome variable of interest is *time until an event occurs*. Because of censoring–the nonobservation of the event of interest after a period of follow-up–a proportion of the survival times of interest will often be unknown. It is assumed that those patients who are censored have the same survival prospects as those who continue to be followed, that is, the censoring is uninformative. Survival data are generally described and modelled in terms of two related functions, the survivor function and the hazard function. The survivor function represents the probability that an individual survives from the time of origin to some time beyond time *t*. It directly describes the survival experience of a study cohort, and is usually estimated by the KM method. The logrank test may be used to test for differences between survival curves for groups, such as treatment arms. The hazard function gives the instantaneous potential of having an event at a time, given survival up to that time. It is used primarily as a diagnostic tool or for specifying a mathematical model for survival analysis. In comparing treatments or prognostic groups in terms of survival, it is often necessary to adjust for patient-related factors that could potentially affect the survival time of a patient. Failure to adjust for confounders may result in spurious effects. Multivariate survival analysis, a form of multiple regression, provides a way of doing this adjustment, and is the subject the next paper in this series.
